# Engineered membrane-coated nanoparticles enhance ferroptosis and microtubule inhibition in prostate cancer

**DOI:** 10.1007/s10856-026-07074-7

**Published:** 2026-05-21

**Authors:** Kunmu Yang, Yingwei Wang, Chuan Guo, Xiaoxiong Zhang, Jian Wu

**Affiliations:** 1 Department of Urology, Chengfei Hospital, Chengdu, China; 2 Department of Nuclear Medicine, Affiliated Cancer Hospital of University of Electronic Science and Technology of China, Chengdu, China

## Abstract

Prostate cancer (PCa), characterized by its high incidence and progression to treatment-resistant stages, remains a major clinical challenge. Herein, we developed a membrane-coated Cabazitaxel (Cab)@TA–Fe³⁺ nanoplatform for enhanced PCa therapy. The nanoparticles demonstrated notable anticancer effects, with cytotoxicity increasing over time and with dose, along with significant inhibition of cell migration and induction of G2/M phase arrest in LNCaP cells. In addition, treatment markedly increased intracellular reactive oxygen species (ROS) levels, depleted glutathione (GSH), downregulated Glutathione Peroxidase 4 (GPX4) expression, and upregulated NADPH Oxidase 1 (NOX1) and Prostaglandin-Endoperoxide Synthase 2 (PTGS2), indicating enhanced ferroptosis. Ferrostatin-1 partially restored cell viability and reduced ROS accumulation, further supporting the involvement of ferroptosis. Mechanistically, the nanoplatform enables pH/GSH-responsive release of Cab and iron ions in the tumor microenvironment, promoting microtubule disruption and redox imbalance. These combined effects enhance tumor cell death. Overall, this study demonstrates that Cab@TA–Fe³⁺ nanoparticles significantly improve anticancer efficacy by integrating microtubule inhibition and ferroptosis induction, providing a promising strategy for prostate cancer treatment.

**Graphical Abstract:**

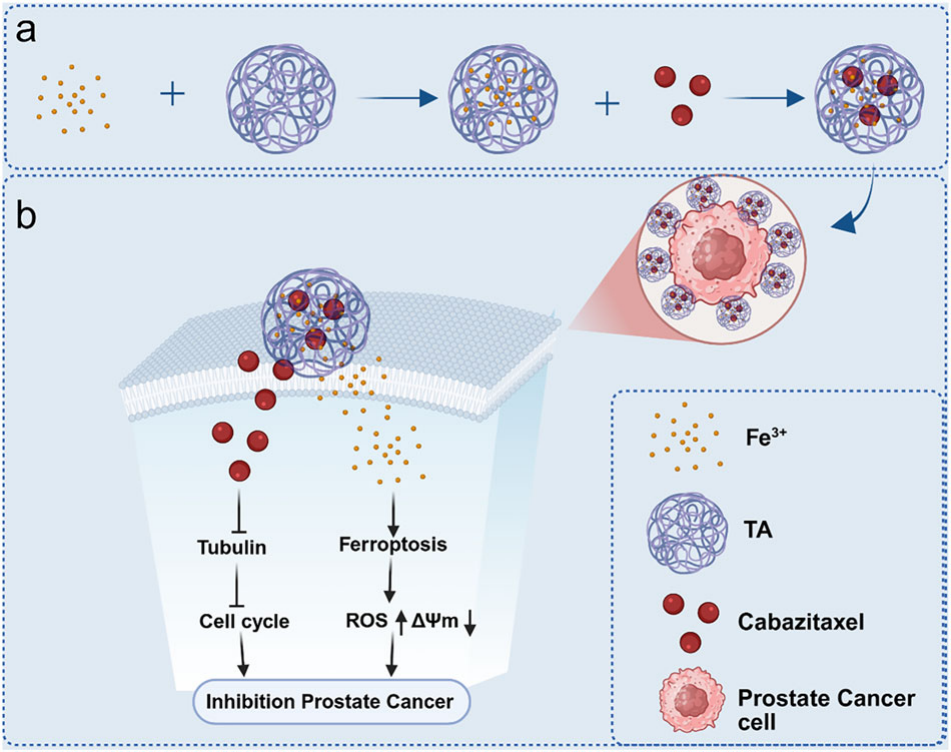

## Introduction

Prostate cancer (PCa) is currently the second most prevalent malignancy among men worldwide and ranks as the fifth leading cause of cancer-related deaths in men [1, 2]. Due to the insidious onset of prostate cancer, early diagnosis is often challenging, and bone metastasis is commonly detected at the time of diagnosis [3, 4]. Most early-stage prostate cancers are androgen-dependent and responsive to endocrine therapy [5]. However, after 12 to 18 months of anti-androgen treatment, the disease often progresses to androgen-independent prostate cancer (hormonal refractory prostate cancer, HRPC), which becomes resistant to endocrine therapies [6]. Even after androgen deprivation, tumors continue to proliferate, leading to disease progression and ultimately resulting in patient mortality [7]. As such, there is an urgent need to develop innovative therapeutic strategies for the effective treatment of PCa.

Tubulin, an essential component of the cytoskeleton, plays a pivotal role in various cellular processes, including mitosis, intracellular transport, and maintenance of cell shape [8]. Moreover, abnormal overexpression of tubulin is frequently observed in many tumor cells, which further contributes to tumorigenesis [9, 10]. As a result, tubulin has become a critical target for the research and development of novel anticancer therapies. Cabazitaxel (Cab) is a second-line treatment for prostate cancer and a synthetic taxane-based antitumor drug [11]. Its antitumor effect primarily arises from its ability to inhibit microtubule disassembly, thereby stabilizing microtubules, which disrupts mitosis and interphase cell functions [12]. Although Cab shows significant effects in cancer treatment, monotherapy is not sufficient for long-term clinical use, prompting researchers to explore combinations with other strategies. Various drug-delivery nanomaterials, such as liposomes, bioceramics, and carbon nanotubes, have been utilized in cancer therapy, with natural nanomaterials and biomaterials gaining attention for their cost-effectiveness, safety, and ability to inhibit cancer cell growth and metastasis [13–15]. Phenolic natural compounds, like tannic acid (TA), are of particular interest due to their non-toxic, stable, antioxidant, and biodegradable properties [16]. TA, a naturally occurring polyphenol, can form a robust metal-phenol network (MPN) with metal ions, making it a promising candidate for use in nanomedicine and cancer therapy as a drug delivery carrier [17]. As a novel biomedical intervention, cellular hitchhiking has emerged as a novel strategy to modulate the biological functions of transplanted immune cells, utilizing engineered micro/nanoscale carriers that are attached to the cell membrane for targeted therapeutic delivery [18]. Recent developments have focused on the application of ferric-based supramolecular coordination complexes, also known as MPN, in membrane engineering to facilitate surface functionalization [19]. MPN-modified erythrocytes have successfully carried adeno-associated viral vectors for genomic alterations, while the conjugation of PD-L1 antibodies to macrophages via MPNs has demonstrated promising outcomes in cancer treatment research [20].

Ferroptosis is a newly identified form of iron-dependent regulated cell death, distinct from apoptosis, autophagy, and necrosis [14]. Studies have shown that ferroptosis can be induced by delivering exogenous iron to tumor sites [21]. This process involves the consumption of glutathione (GSH) by Fe^3+^, which is converted into oxidized glutathione disulfide, leading to the inactivation of Glutathione Peroxidase 4 (GPX4) and ultimately resulting in ferroptotic cell death [22, 23]. Therefore, exogenous delivery of Fe^3+^ to deplete GSH and convert it into Fe^2+^ may represent a promising strategy for inducing ferroptosis in tumor treatment. We hypothesize that combining ferroptosis with microtubule targeting may represent an effective combined strategy for inducing tumor cell death.

Building on the studies outlined above, we developed a system that employs the coordination self-assembly of TA with Fe³⁺ to form a MPN that encapsulates Cab through hydrophobic interactions, referred to as membrane-coated Cab@TA-Fe³⁺ nanoparticle. Here, “membrane-coated” refers to the adhesion of TA–Fe³⁺ MPNs onto the cell surface, rather than biomimetic membrane encapsulation. This system operates on a pH/GSH dual-response release mechanism, where the acidic tumor microenvironment triggers the dissociation of the TA-Fe³⁺ complex, and elevated GSH levels reduce Fe³⁺ to Fe²⁺, facilitating the concurrent release of Cab and iron ions. We found that membrane-coated Cab@TA-Fe³⁺ complex enhances anti-tumor efficacy through several mechanisms. It induces cytotoxicity, causes G2/M phase cell cycle arrest, disrupts microtubule integrity, inhibits cell migration, and promotes apoptosis. Additionally, Cab@TA-Fe³⁺ triggers ferroptosis by increasing reactive oxygen species (ROS) accumulation, reducing GSH levels, downregulating GPX4 expression, and upregulating NOX1 and PTGS2. These effects amplify oxidative stress, promote lipid peroxidation, and increase the tumor cells’ sensitivity to ferroptosis, further strengthening its therapeutic potential.

## Experimental section

### Materials

TA and FeCl_3_·6H_2_O were purchased from Sigma-Aldrich (St. Louis, MO, USA). Cab was obtained from Dalian Meilun Biotech Co., Ltd (Dalian, China). Primary monoclonal antibodies (Glutathione, Tubulin and GPX4) and secondary antibodies were purchased from HuaBio (Huangzhou, China). BSA-FITC was obtained from Solarbio (Beijing, China). Human Prostate Cancer Cells (LNCaP), Cell Cycle and Apoptosis Analysis Kit, Reactive Oxygen Species Assay Kit and Mitochondrial membrane potential assay kit with JC-1 were bought from Beyotime (Shanghai, China). Dulbecco’s modified Eagle’s medium (DMEM) medium, fetal bovine serum (FBS), penicillin-streptomycin and phosphate-buffered saline (PBS) were purchased from Life Technologies (Carlsbad, CA). CCK-8 reagent, Crystal violet staining solution, DAPI, 4% paraformaldehyde, and tris buffered saline tween (TBST) were purchased from Biosharp (Hefei, China). TRIzol, RNA Extraction Kit, Reverse Transcription Kit, and SYBR Green Master Mix were purchased from Takara (Japan).

### Patient specimens

Prostate cancer (PCa) tissues and benign prostatic hyperplasia (BPH) tissues were obtained from patients who underwent surgical procedures at Chengfei hospital in 2024. A total of 3 PCa samples and 3 BPH samples were included in this study. The diagnosis of PCa and BPH was confirmed by experienced pathologists based on histopathological evaluation. Patients who had received chemotherapy, radiotherapy, or other anticancer treatments prior to surgery were excluded. All tissue specimens were collected immediately after surgical resection and either fixed in formalin for histological analysis. This study was approved by the Institutional Ethics Committee of Chengfei Hospital (approval number: 2024079), and written informed consent was obtained from all participants.

### Histological analysis

Tissue Section (4 μm thick) were rehydrated and subjected to antigen retrieval in citrate buffer at 90°C for 5 min. After blocking with 2% FBS in 0.2% TBS-Triton, sections were incubated overnight at 4°C with primary antibodies against Glutathione (1:100, HuaBio, China) and Tubulin (1:100, HuaBio, China). After washing with TBS, sections were incubated with biotinylated donkey anti-rabbit IgG (1:500), followed by streptavidin–peroxidase (1:500). Signals were visualized using DAB. For quantification, images were captured from at least five randomly selected non-overlapping fields per section under identical settings. The staining intensity was analyzed using ImageJ software. Data are presented as the mean value per sample, and *n* = 3 represents three independent biological samples (tumor tissues) per group.

### Preparation of Cab@TA-Fe^3+^ nanoparticles

All solutions were freshly prepared and filtered through 0.22 μm pore filters prior to use. Cab powder was dissolved in ethanol at concentrations of 2.5, 5, and 10 mg/mL. Briefly, 1 mL of Cab solution at each concentration was rapidly injected into 97 mL of Milli-Q water under ultrasonication and sonicated for 2 min to form a uniform dispersion. Subsequently, 1 mL of tannic acid (TA) solution (20, 40, and 80 mg/mL) and 1 mL of FeCl₃ solution (10 mg/mL) were immediately added. The resulting nanoparticles were collected and washed with distilled water to remove excess Fe³⁺ ions and unbound TA. Unless otherwise specified, all procedures were performed at room temperature under ambient conditions.

### Characterization of Cab@TA-Fe^3+^ nanoparticles

Morphological features and elemental mapping analysis of Cab@TA-Fe^3+^ nanoparticles were analyzed by scanning electron microscopy (SEM, Olympus, 5 kV acceleration voltage). The chemical composition of Cab@TA-Fe^3+^ nanoparticle was characterized using X-ray photoelectron spectroscopy (XPS, Thermo Scientific, UK). The hydrodynamic diameter distribution of Cab@TA-Fe^3+^ nanoparticles dispersed in deionized water was assessed through dynamic light scattering (DLS) measurements (Brookhaven Instruments Corporation). The cells were coated with Cab@TA-Fe³⁺ via surface adhesion of TA–Fe³⁺ MPNs, and subsequently incubated with a FITC-conjugated bovine serum albumin solution (BSA-FITC, 0.2 mg/mL) for 30 min. Following this, Hoechst staining was applied for a duration of 10 min. Fluorescent images were then acquired using a confocal laser scanning microscope (CLSM, Olympus FV3000, Japan). The ultravioletvisible (UV-Vis) spectra of TA, Fe^3+^, and Cab@TA-Fe³⁺ were measured using UV (Shimadzu, Kyoto, Japan). The release of Fe^3+^ was detected using inductively coupled plasma mass spectrometry (iCAP 6300, Thermo Fisher Scientific; USA). To investigate the release kinetics of Fe³⁺ at PH = 5.5 and PH = 7.4, supernatants were gathered after culturing Cab@TA-Fe³⁺ for 24, 48, 72, and 96 h. These samples were then analyzed using inductively coupled plasma mass spectrometry (iCAP 6300, Thermo Fisher Scientific, USA). The obtained data were compiled and analyzed using GraphPad software. To assess the drug release from both free Cab and Cab@TA-Fe³⁺, a dialysis method was employed. Dialysis bags, each containing 1 mL of free Cab or Cab@TA-Fe³⁺ (with a molecular weight cut-off of 1000 Da), were immersed in 10 mL of PBS (pH 5.5 and 7.4) supplemented with 0.05% (V/V) Tween 20. The beakers were placed on a shaker, and at specific time intervals, 2 mL of the dialysate was withdrawn and replaced with fresh buffer. The released Cab was quantified by high-performance liquid chromatography (Waters E2695, Milford, MA, USA).

### Cell culture

Human prostate cancer cells (LNCaP) were routinely maintained in Dulbecco’s modified Eagle’s medium (DMEM) supplemented with 10% fetal bovine serum, 100 U/mL penicillin, and 100 μg/mL streptomycin, unless they were cultured in serum-free medium for transwell experiments. The cells were incubated at 37°C in a humidified environment containing 5% CO_2_.

### Cell counting kit- 8 (CCK- 8) assay

LNCaP cells were plated in culture plates with 96 wells at 1×10⁴ cells per well, then exposed to Cab@TA-Fe³⁺ (Cab-equivalent doses of 0.25, 0.50, and 1.00 mg·mL⁻¹), TA-Fe³⁺, or Cab in its unencapsulated form. At the 24-, 48-, and 96-h time points, a 10-ml aliquot of CCK-8 reagent was added to each well and incubation continued for an additional 1 to 4 h. Absorbance was subsequently read at a wavelength of four hundred and fifty nanometers, and cell viability was calculated by normalizing each reading to the untreated control group.

### Cell cycle assay

Cell-cycle distribution was assessed by flow cytometry using a commercial analysis kit (Beyotime). After a 24-h exposure to Cab@TA-Fe³⁺, TA-Fe³⁺, or free Cab, LNCaP cells were detached with 0.25% trypsin and collected by centrifugation (1000 rpm, 5 min). The pellets were then resuspended in 1 mL of ice-cold 70% ethanol and fixed at 4 °C for 24 h. Following a second centrifugation, each sample was incubated with 500 µL propidium iodide solution for 30 min at 37 °C in the dark. Events were acquired on a CytoFLEX flow cytometer (BECKMAN, USA), and cell-cycle profiles were quantified with Graphpad10.0 software.

### Cell migration assay

Transwell migration of LNCaP cells was assessed using 24-well Falcon permeable inserts (Corning Inc., Corning, NY, USA). After pretreatment with Cab@TA-Fe³⁺, TA-Fe³⁺, or free Cab, cell suspensions were seeded into the upper chamber in serum-free DMEM. The lower chamber was also filled with serum-free DMEM to assess basal cell migration in the absence of a chemotactic gradient. After 24 h of incubation, cells were fixed with 4% paraformaldehyde for 15 min at room temperature, washed with PBS, and stained with 0.1% crystal violet for 5 min. Cells that migrated to the underside of the membrane were imaged using an inverted microscope (Olympus, Tokyo, Japan) and quantified using ImageJ.

### Immunofluorescence staining

After a 24-h exposure to Cab@TA-Fe³⁺, TA-Fe³⁺, or free Cab, LNCaP cells were rinsed three times with phosphate-buffered saline (PBS) and crosslinked with 4% paraformaldehyde (PFA) for 15 min at ambient temperature. Membranes were then permeabilized with 0.25% (v/v) Triton X-100 for 10 min at room temperature, followed by additional PBS rinses. To reduce nonspecific binding, specimens were blocked in goat serum for 30 min at room temperature. Primary antibodies (anti-Tubulin and anti-Glutathione; HuaBio; 1:100) were applied overnight at 4 °C. Afterward, samples were washed three times with TBST, incubated with the appropriate secondary antibody for 60 min at room temperature, and nuclei were counterstained with DAPI for 5 min in the dark. Images were acquired on a confocal laser scanning microscope (Olympus FV3000, Japan), and fluorescence signals were quantified using ImageJ.

### Reactive oxygen species (ROS) detection

LNCaP cells were seeded in 24-well plates at a density of 1×10⁵ cells per well and treated with Cab@TA-Fe³⁺, TA-Fe³⁺, or free Cab for 8 h. Following three washes with PBS, the cells were incubated with 10 μM DCFH-DA for 30 min to assess ROS levels. Afterward, the cells were washed again with PBS three times. Fluorescence images were taken using a microscope (Olympus, Japan), and the stained regions were quantified using ImageJ.

### Mitochondrial membrane potential (MMP) detection

JC-1 is a lipophilic, cationic dye that selectively accumulates in the inner mitochondrial membrane, reflecting mitochondrial membrane potential (MMP). In intact mitochondria, it forms red fluorescent aggregates, whereas in depolarized mitochondria, it shifts to green fluorescent monomers. Cells were incubated with JC-1 for 30 min at 37 °C. Fluorescent images were captured using a microscope (Olympus, Japan), and the stained areas were analyzed with ImageJ software. The red/green fluorescence intensity ratio was used as a semi-quantitative measure of MMP.

### Quantitative reverse transcription PCR (qPCR)

qPCR was performed to assess the expression of genes related to ferroptosis. LNCaP cells were seeded in 6-well plates at a density of 2×10⁵ cells per well and treated with Cab@TA-Fe³⁺, TA-Fe³⁺, or free Cab for 24 h. Afterward, total RNA was extracted using an RNA Isolation Kit (Takara, Japan), and its concentration was measured using a microplate reader. Full- length complementary DNA (cDNA) was synthesized using a Reverse Transcription Kit (Takara, Japan). qPCR was carried out with SYBR Green Master Mix on a Roche LightCycler 96 instrument (Roche, Switzerland), employing glyceraldehyde-3- phosphate dehydrogenase (GAPDH) as the internal control gene for normalization. The data were analyzed using the 2^−△△ct^ method, and the primer sequences are provided in Table S1.

### Ferroptosis inhibition assay

To investigate the potential involvement of ferroptosis, a rescue experiment was performed using Ferrostatin-1 (Fer-1, MCE, USA). Cells were seeded in 96-well plates and incubated overnight. Subsequently, cells were treated with Cab@TA–Fe³⁺ nanoparticles in the presence or absence of Fer-1 (2 μM) for 24, 48, or 96 h. Cell viability was assessed using the CCK-8 assay according to the manufacturer’s instructions. For intracellular ROS detection, cells were incubated with DCFH-DA at 37 °C for 30 min, followed by washing with PBS. Fluorescence images were obtained using a fluorescence microscope, and the mean fluorescence intensity was quantified using ImageJ software.

## Results

### Elevated expression of glutathione and tubulin in prostate cancer tissues

**Fig. 1:**
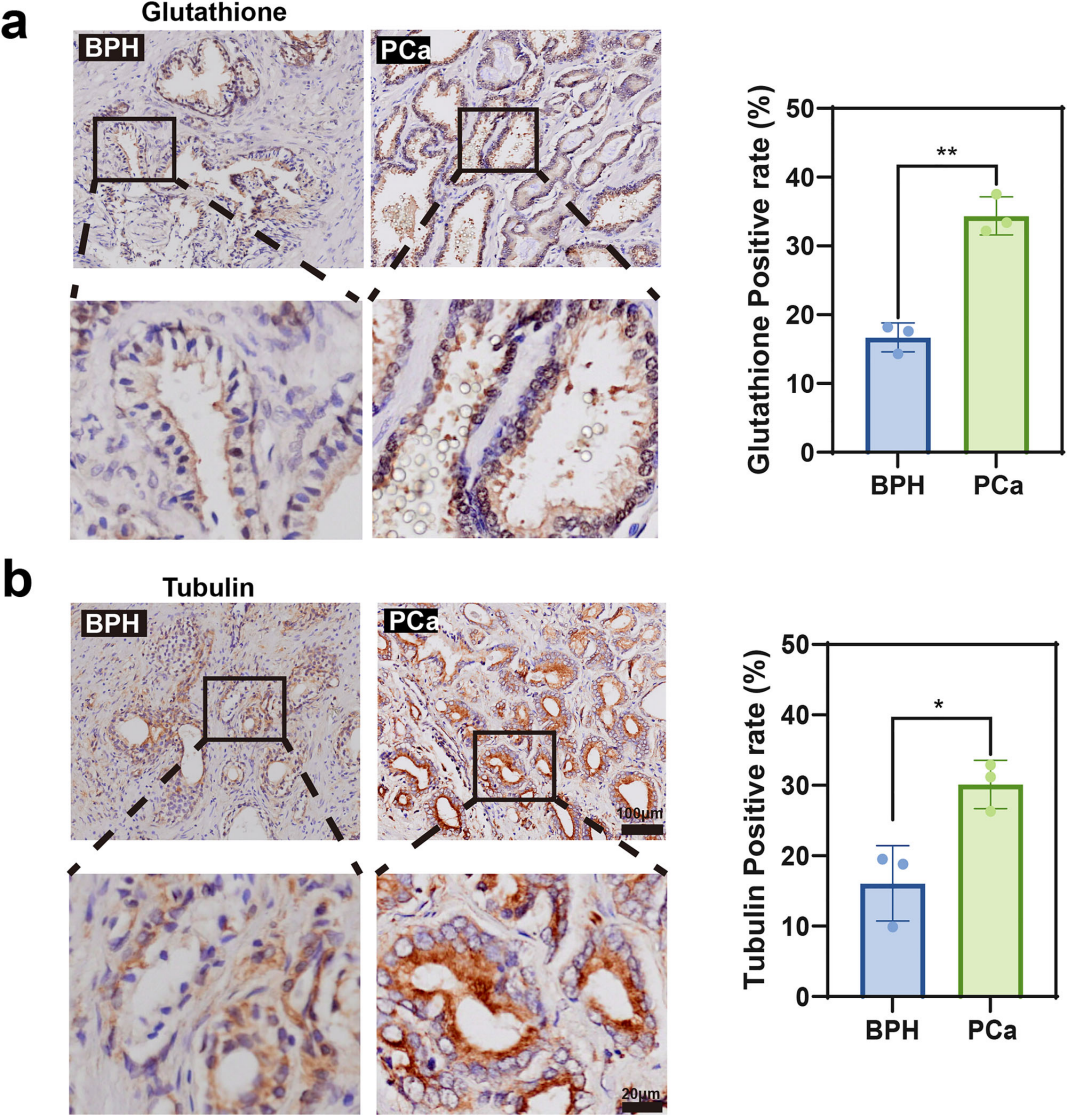
Immunohistochemical (IHC) analysis of glutathione and tubulin expression in benign prostatic hyperplasia (BPH) and prostate cancer (PCa) tissues. **a** Representative IHC staining images of glutathione in BPH and PCa tissues (upper panel, scale bar: 100 µm; lower panel, enlarged views, scale bar: 20 µm). Quantification of glutathione-positive rates reveals a significant upregulation in Pca tissues compared to BPH (**p < 0.01). **b** Representative IHC staining images of tubulin in BPH and PCa tissues (upper panel, scale bar: 100 µm; lower panel, enlarged views, scale bar: 20 µm). Quantification indicates a markedly elevated tubulin-positive rate in PCa tissues relative to BPH (*p < 0.05). Data are presented as mean ± SD (*n* = 3), and statistical significance was determined using unpaired two-tailed Student’s t-test

Immunohistochemical (IHC) staining was performed to assess the expression of GSH and tubulin in PCa and benign prostatic hyperplasia (BPH) tissues. Representative images are shown in Fig. 1. Quantitative analysis revealed significantly higher GSH-positive staining in Pca tissues compared with BPH samples (*p* < 0.01) (Fig. 1a**)**. Similarly, tubulin expression was markedly increased in PCa tissues relative to BPH controls (*p* < 0.05) (Fig. 1b**)**. These findings suggest that elevated glutathione and tubulin expression may jointly contribute to the malignant phenotype of prostate cancer by promoting tumor growth, therapeutic resistance, and invasive potential. Such co-upregulation highlights both molecules as potential biomarkers and therapeutic targets for more effective prostate cancer management.

### Fabrication and characterization of Cab@TA-Fe^3+^

**Fig. 2:**
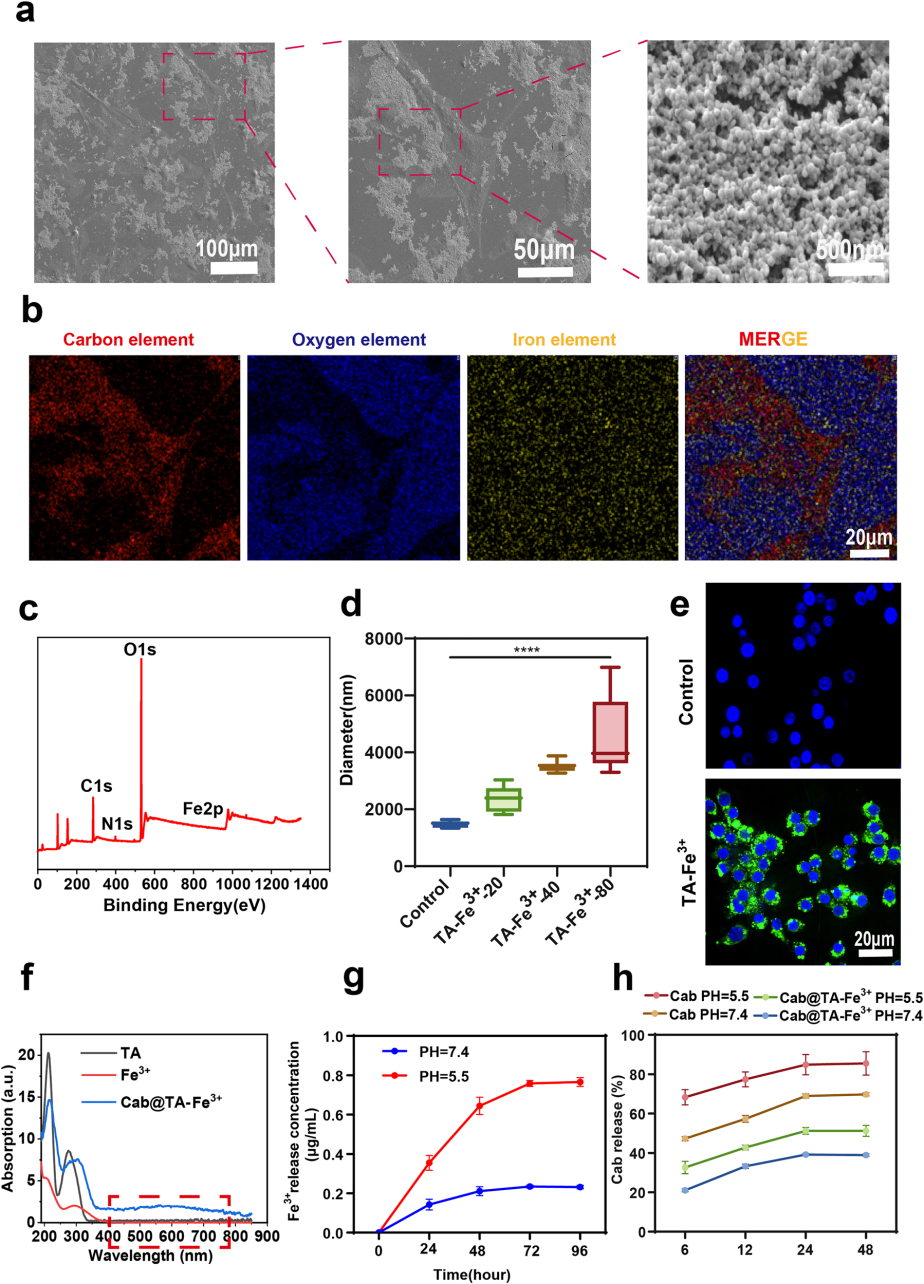
**a** Fabrication and characterization of Cab@TA-Fe^3+^. Scanning electron microscopy (SEM) images showing the morphology and size of Cab@TA-Fe³⁺ (scale bars: 100 µm, 50 µm, and 500 nm). **b** Elemental mapping images of Cab@TA-Fe³⁺ complex (scale bar: 20 µm). **c** XPS survey spectrum of the Cab@TA-Fe³⁺. **d** Dynamic light scattering (DLS) characterization for the sizes of Cab@TA-Fe³⁺ with different concentrations (20 mg/mL, 40 mg/mL, and 80 mg/mL), (****p < 0.0001). **e** Representative fluorescent images of Cab@TA-Fe³⁺ coating, scale bar = 20 μm. **f** UV–vis absorption spectra of TA, Fe³⁺, and Cab@TA–Fe³⁺ nanoparticles. **g** Cumulative Fe release from the Cab@TA-Fe³⁺ coating over time at different pH values (7.4 and 5.5). **h** The release kinetics of cabazitaxel (Cab) from Cab@TA-Fe³⁺ nanocomposite system under both neutral (pH 7.4) and acidic (pH 5.5) conditions

To design a system capable of consuming glutathione and targeting tubulin, we employed the coordination self-assembly of tannic acid (TA) with Fe³⁺ to form a MPN. This network is presumed to encapsulate Cab primarily through hydrophobic interactions, consistent with previous reports on polyphenol-based nanoplatforms for hydrophobic drug loading hydrophobic interaction [24]. In the acidic tumor microenvironment, the coordination between TA and Fe³⁺ is destabilized under low pH conditions, leading to partial dissociation of the metal–phenol network metal–phenol coordination [25]. Meanwhile, the elevated intracellular GSH levels in tumor cells can reduce Fe³⁺ to Fe²⁺, promoting redox cycling and facilitating the release of both Cab and iron ions redox reaction [26]. This dual-responsive behavior has been widely reported in metal–phenol-based nanoplatforms [27]. Scanning electron microscopy (SEM) demonstrated a porous, interconnected network morphology characteristic of metal–phenol coordination architectures (Fig. 2a). Elemental mapping analysis confirmed uniform distribution of carbon, oxygen, and iron elements throughout the nanostructure (Fig. 2b). X-ray photoelectron spectroscopy (XPS) further verified successful incorporation of Fe³⁺, with characteristic Fe2p, C1s, O1s, and N1s peaks detected in the survey spectrum (Fig. 2c). Dynamic light scattering (DLS) analysis showed concentration-dependent size variation (Fig. 2d). Drug-free TA–Fe³⁺ nanoparticles prepared with Fe³⁺ (10 mg/mL) and TA (40 mg/mL) exhibited optimal size distribution and stability, and these conditions were subsequently used for the preparation of Cab-loaded nanoparticles. Fluorescence imaging confirmed successful surface coating of TA–Fe³⁺ complexes on prostate cancer cells (Fig. 2e). UV–vis absorption spectroscopy was employed to investigate the formation of the nanoparticles (Fig. 2f). TA and Fe³⁺ exhibited characteristic absorption peaks in the UV region. After coordination, the TA–Fe³⁺ complex showed a broadened absorption profile, indicating the formation of metal–phenolic coordination structures. Notably, the Cab@TA–Fe³⁺ nanoparticles displayed a distinct absorption change compared with TA–Fe³⁺, suggesting successful encapsulation of Cab within the network, which may be attributed to intermolecular interactions such as hydrophobic interactions and π–π stacking. Drug release studies demonstrated minimal Fe³⁺ and Cab release under physiological conditions (pH 7.4), whereas rapid release was observed under acidic conditions (pH 5.5) (Fig. 2g, h). The results highlight that the pH/GSH dual-response release mechanism effectively enables the targeted and controlled release of Cab and Fe³⁺ within the acidic environment of the tumor microenvironment.

### Cab@TA-Fe^3+^ induces cell death through microtubule inhibition in LNCaP cells

**Fig. 3:**
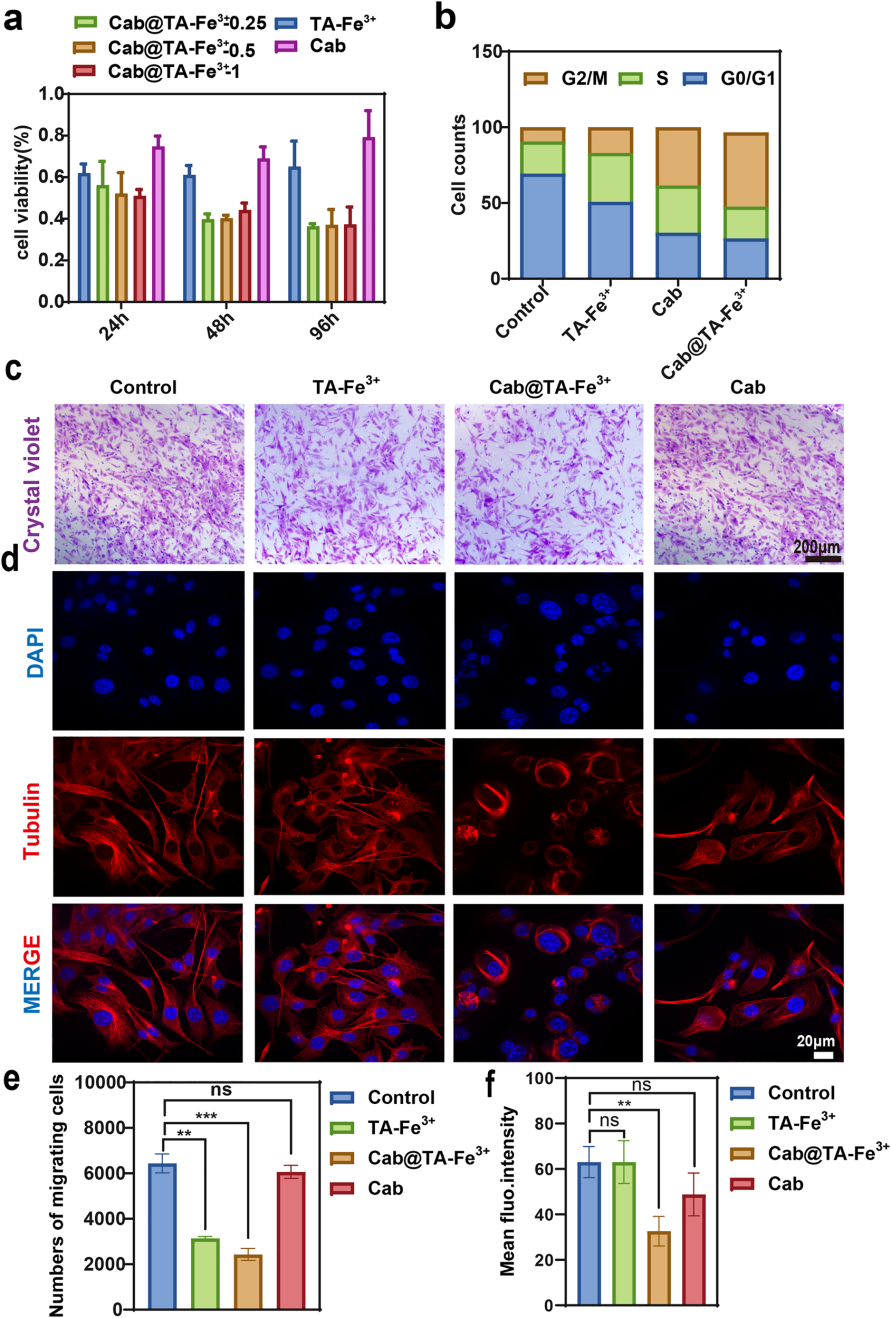
**a** In vitro anti-tumor performance of Cab@TA–Fe³⁺. Cell viability of LNCaP cell after incubation with different concentrations of Cab@TA-Fe³⁺ (Cab: 0.25 mg/mL, 0.5 mg/mL, and 1 mg/mL), TA-Fe³⁺, and Cab for 24, 48, and 96 h, as determined by CCK-8 assay. **b** Flow cytometry analysis of cell cycle distribution in LNCaP cells. **c** Representative crystal violet staining images illustrating the migration of LNCaP cells. Scale bar: 200 μm. **d** Immunofluorescence staining of α-tubulin (red) and nuclei (DAPI, blue) in LNCaP cells. Scale bar: 20 μm. **e** Quantification of migrated LNCaP cells in a transwell migration assay. **f** Quantitative analysis of intracellular fluorescence intensity. Data are presented as mean ± SD (*n* = 3), **p < 0.01, ***p < 0.001, ns: not significant

The membrane-coated (i.e., MPN-adhered) Cab@TA-Fe^3+^ complex can efficiently release both Cab and Fe^3+^ in the acidic tumor microenvironment, thereby enhancing cellular uptake of Cab and iron ions. Subsequent investigations were conducted to further evaluate the anti-tumor efficacy. To assess the anti-tumor efficacy of Cab@TA-Fe³⁺ in vitro, we first evaluated the viability of LNCaP cells upon exposure to various concentrations of Cab@TA-Fe³⁺(Cab: 0.25 mg/mL, 0.5 mg/mL, and 1 mg/mL), TA-Fe^3+^, or free Cab for 24, 48, and 96 h. As shown in Fig. 3a, Cab@TA-Fe³⁺ exhibited a pronounced time- and dose-dependent cytotoxicity, markedly surpassing that of TA-Fe^3+^ or Cab alone, suggesting an enhanced combined effect between Cab and Fe³⁺. The Cab concentration of 0.5 mg/mL in Cab@TA–Fe³⁺ exhibited the most pronounced inhibitory effect on tumor growth, and this concentration was therefore selected for subsequent experiments. Flow cytometric analysis further revealed that Cab@TA-Fe³⁺ treatment induced a significant G2/M phase arrest (Fig. 3b), indicating effective disruption of cell cycle progression. The results showed that membrane-coated Cab@TA-Fe³⁺ complex decreased the percentage of cells in the G0/ G1 phase. This interference disrupts the normal dynamic behavior of microtubules, leading to cell cycle arrest at the G2/M phase and subsequently promoting cell death. Next, the migration ability of the LNCaP cells was evaluated using transwell. The results showed that Cab@TA-Fe³⁺ dramatically suppressed cell migration, with the number of migrating cells significantly lower than that in the TA-Fe³⁺ or Cab groups (Fig. 3c, e). The membrane-coated Cab@TA-Fe³⁺ complex interacts with microtubules, ultimately inducing cell death. In comparison to the control group, the Cab@TA-Fe³⁺ complex significantly disrupted microtubule integrity, leading to the degradation of the microtubule network and the loss of filamentous structures (Fig. 3d, f). These findings suggest an enhanced combined effect between Cab and Fe³⁺, where the combination of Cab and Fe³⁺ not only induces significant cell cycle arrest but also disrupts the microtubule network, impairing cell migration and promoting death.

### Cab@TA–Fe^3+^ induces ferroptosis-associated redox dysregulation

**Fig. 4:**
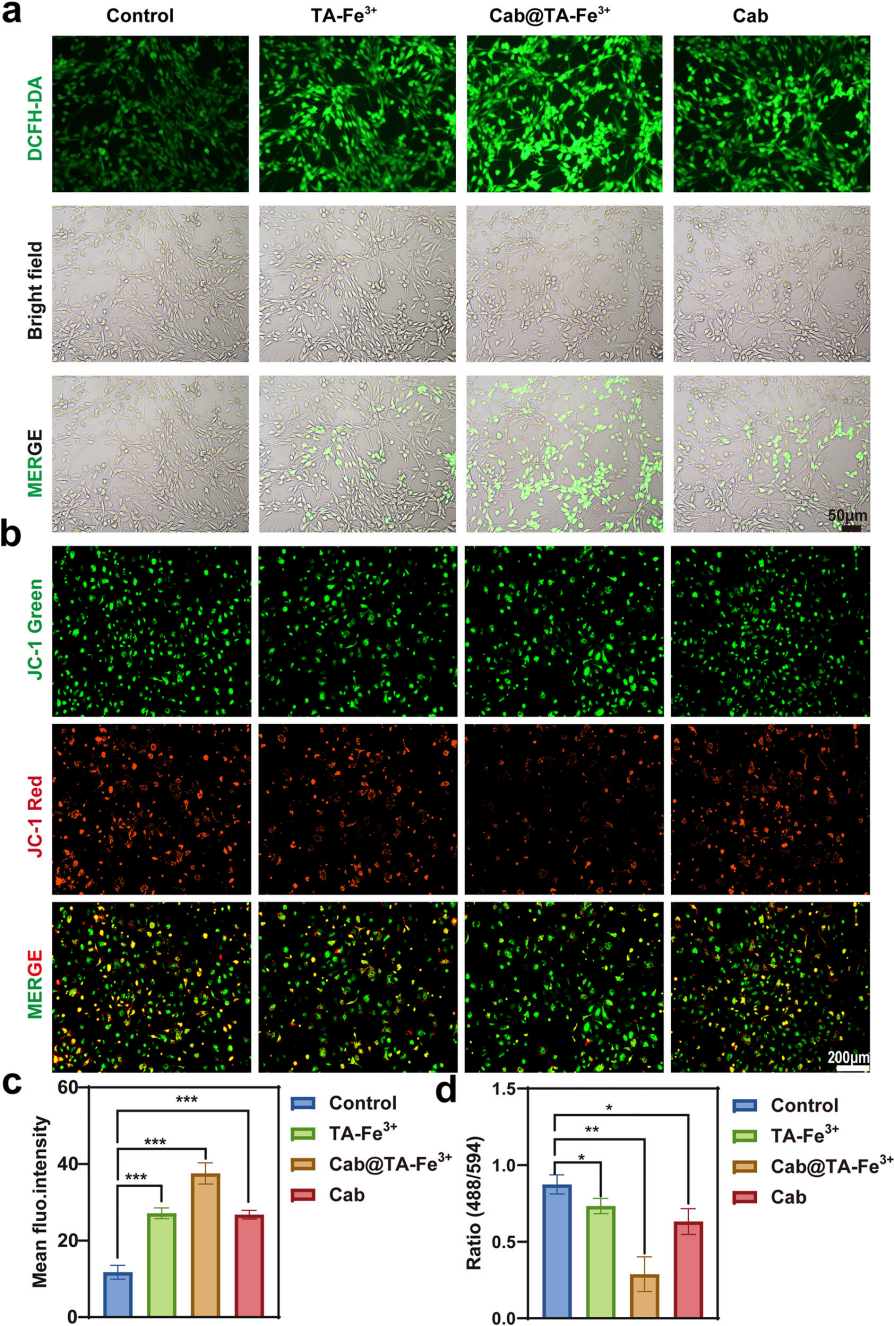
**a** Evaluation of oxidative stress and mitochondrial membrane potential after treatment with Cab@TA-Fe³⁺. The levels of reactive oxygen species (ROS) were assessed in LNCaP cells using DCFH-DA staining. **b** Mitochondrial membrane potential was measured using JC-1 staining. Green fluorescence indicates a depolarized mitochondrial state, while red fluorescence indicates a polarized state. **c** Quantification of the mean fluorescence intensity from ROS assays. **d** Quantification of green/red fluorescence ratio. Data are presented as mean ± SD (*n* = 3), *p < 0.05, **p < 0.01, ***p < 0.001

**Fig. 5:**
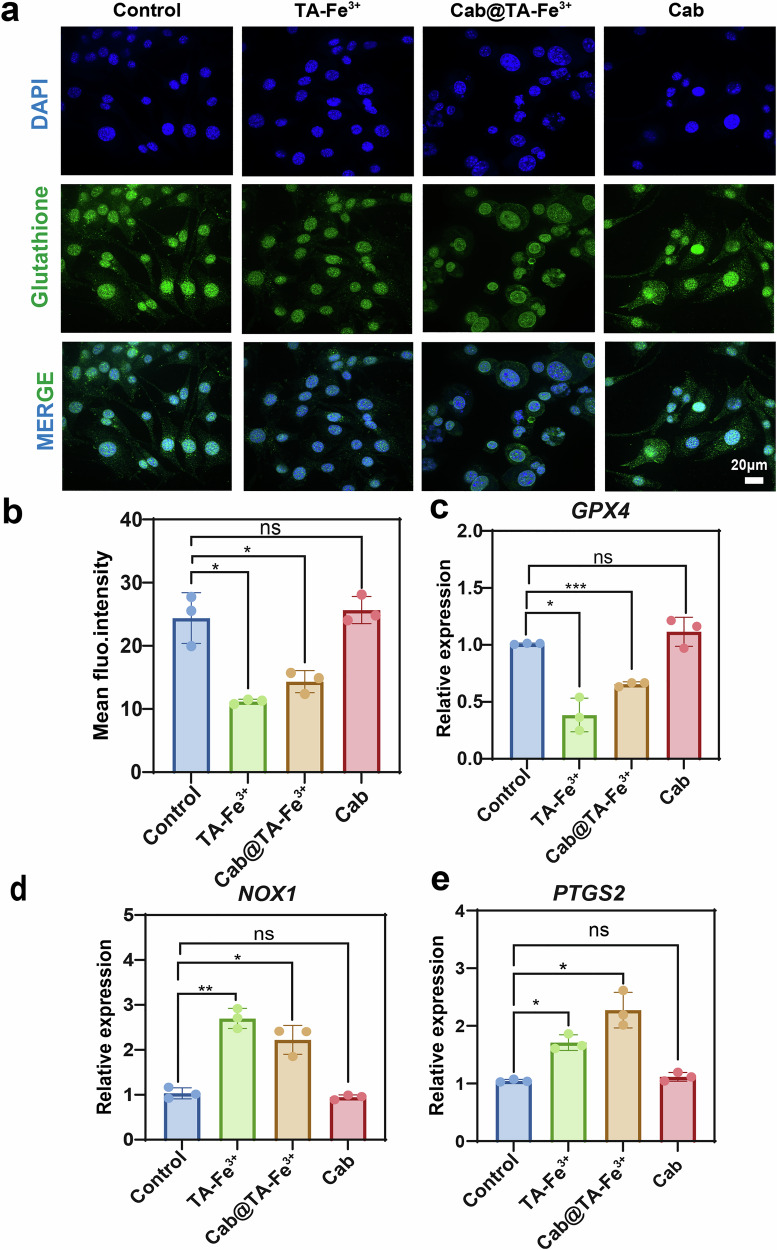
**a** Evaluation of glutathione (GSH) levels and genes related to ferroptosis in response to Cab@TA-Fe³⁺ treatment. Fluorescence microscopy images showing GSH levels in LNCaP cells treated with different formulations (Control, TA-Fe³⁺, Cab@TA-Fe³⁺, and Cab). **b** Quantification of mean fluorescence intensity of glutathione levels. **c** The relative expression of GPX4 in control, TA-Fe³⁺, Cab@TA-Fe³⁺, and Cab group. **d** The relative expression of NOX1 in control, TA-Fe³⁺, Cab@TA-Fe³⁺, and Cab group. **e** The relative expression of PTGS2 in control, TA-Fe³⁺, Cab@TA-Fe³⁺, and Cab group. Data are presented as mean ± SD (*n* = 3), *p < 0.05, **p < 0.01, ***p < 0.001, ns: not significant

ROS levels were quantified using DCFH-DA staining. Cab@TA–Fe³⁺ treatment significantly increased ROS generation relative to control and comparator groups (Fig. 4a, c). MMP was assessed via JC-1 staining. Treatment with Cab@TA–Fe³⁺ resulted in an increased green/red fluorescence ratio, indicating mitochondrial depolarization, which reflects cellular oxidative stress but is not specific to ferroptosis (Fig. 4b, d). Therefore, additional ferroptosis-specific markers were further evaluated to clarify the underlying cell death mechanism. Fluorescence-based analysis demonstrated significant depletion of intracellular glutathione (GSH) in the Cab@TA–Fe³⁺ group (Fig. 5a, b). Quantitative q-PCR analysis further showed downregulation of GPX4 expression and upregulation of NOX1 and PTGS2 following Cab@TA–Fe³⁺ treatment (Fig. 5c–e). Our results indicate that Cab@TA-Fe³⁺ treatment significantly reduces GSH levels and decreases the expression of GPX4, promoting lipid peroxidation and ferroptosis. Additionally, the upregulation of NOX1 and PTGS2 in treated cells further amplifies oxidative stress and inflammation, collectively enhancing the sensitivity of cells to ferroptosis.

**Fig. 6:**
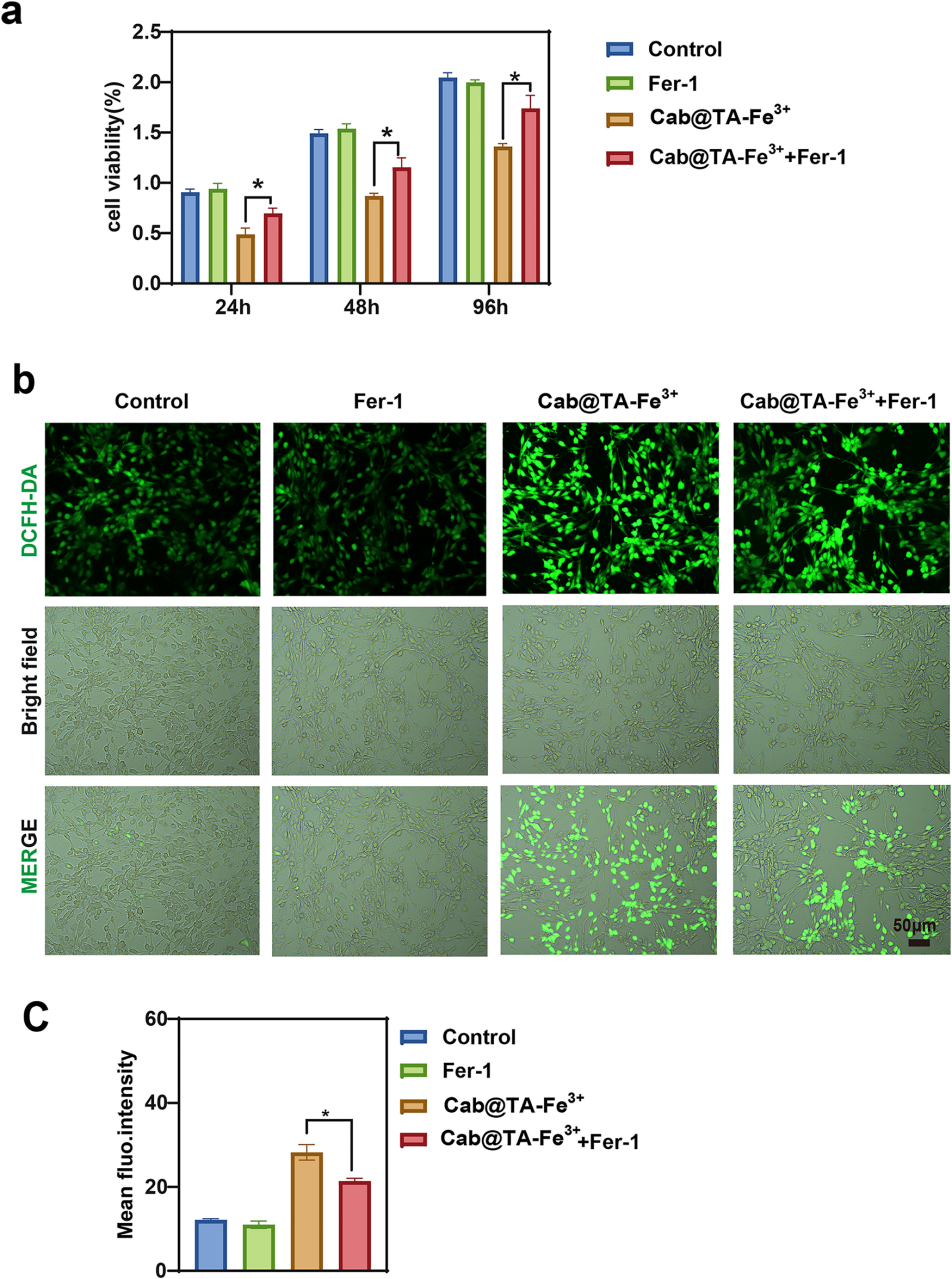
**a** Effects of Ferrostatin-1 (Fer-1) on cell viability and intracellular ROS levels. Cell viability of cells treated with Control, Fer-1, Cab@TA–Fe³⁺, and Cab@TA–Fe³⁺ + Fer-1 for 24, 48, and 96 h. **b** Representative fluorescence images of intracellular ROS levels detected by DCFH-DA staining under different treatments. Bright-field and merged images are also shown. Scale bar: 50 μm. **c** Quantification of the mean fluorescence intensity from ROS assays. Data are presented as mean ± SD. *p < 0.05

To further examine the potential involvement of ferroptosis in the therapeutic mechanism, a ferroptosis inhibition experiment was performed using Ferrostatin-1 (Fer-1). As shown in Fig. 6a, treatment with Cab@TA–Fe³⁺ nanoparticles resulted in a pronounced decrease in cell viability compared with the control group. Notably, co-treatment with Fer-1 partially restored cell viability, suggesting that ferroptosis may contribute to the observed cytotoxicity. In addition, intracellular ROS levels were assessed using DCFH-DA staining **(**Fig. 6b**)**. Cells treated with Cab@TA–Fe³⁺ exhibited enhanced fluorescence signals, indicating increased ROS generation. In contrast, Fer-1 co-treatment attenuated ROS accumulation, which was further supported by quantitative fluorescence analysis **(**Fig. 6c**)**. Taken together, while ROS accumulation and mitochondrial depolarization indicate enhanced oxidative stress, the concurrent GSH depletion, GPX4 downregulation, upregulation of NOX1/PTGS2, and partial rescue by Ferrostatin-1 collectively support the involvement of ferroptosis rather than nonspecific oxidative damage alone.

## Discussion

The innovative aspect of this study lies in the integration of a MPN platform with dual targeting of microtubules and ferroptosis. While cabazitaxel is an established second-line therapy for advanced prostate cancer, its clinical efficacy remains limited by resistance and incomplete tumor eradication. Previous strategies have largely focused on improving drug delivery efficiency or combining Cab with other chemotherapeutic agents. In contrast, the present work combines microtubule inhibition with ferroptosis induction within a single stimulus-responsive nanoplatform. Unlike conventional polymeric or liposomal carriers, the TA–Fe³⁺ coordination framework serves not only as a structural carrier but also as a functional source of redox-active iron. This dual-function design distinguishes the current system from previously reported Cab-based nanocarriers that rely solely on passive delivery.

Compared with conventional chemotherapy, cabazitaxel has demonstrated significant antitumor activity in prostate cancer, particularly in docetaxel-resistant cases. However, its clinical benefit remains limited, with only modest improvements in overall survival (typically 2–4 months), and is often accompanied by systemic toxicity [12, 28, 29]. In recent years, nanoparticle-based delivery systems have been developed to enhance the therapeutic efficacy of cabazitaxel. For example, cabazitaxel nanodispersions and prodrug-based nanoplatforms have shown improved tumor inhibition and reduced side effects compared with free drug formulations [30]. Moreover, emerging studies have demonstrated that ferroptosis-based strategies can significantly enhance antitumor efficacy in prostate cancer by promoting iron-dependent oxidative damage [31]. In comparison with these studies, our Cab@TA–Fe³⁺ nanoparticle system integrates chemotherapy with ferroptosis induction, resulting in enhanced ROS generation, increased lipid peroxidation, and improved tumor inhibition. This dual therapeutic mechanism may account for the superior anticancer efficacy observed in our study.

Iron-based nanoparticles have been widely explored to induce ferroptosis by increasing intracellular iron and ROS levels [15, 32, 33]. For example, functionalized iron oxide nanoparticles and ferritin-based systems have demonstrated ferroptotic effects in various cancers. However, most of these systems primarily rely on iron-mediated oxidative stress without targeting structural vulnerabilities of tumor cells [34, 35]. Compared with previously reported iron-based ferroptosis platforms, the Cab@TA–Fe³⁺ system introduces a combinatorial mechanism. Here, iron-induced redox imbalance is coupled with microtubule stabilization–mediated mitotic arrest. The observed GSH depletion and GPX4 downregulation align with established ferroptosis signatures reported in the literature [36, 37], yet the simultaneous disruption of α-tubulin organization provides an additional cytoskeletal stress component. Thus, rather than functioning as a single-mode ferroptosis inducer, this platform integrates metabolic and structural stress pathways. To further examine the role of ferroptosis, a rescue experiment using Fer-1 was performed. Fer-1 treatment partially restored cell viability and attenuated intracellular ROS levels, suggesting that ferroptosis may contribute to the cytotoxic effects of Cab@TA–Fe³⁺ nanoparticles. These findings are consistent with the observed GSH depletion and GPX4 downregulation. Notably, the incomplete rescue effect indicates that ferroptosis is unlikely to be the sole mechanism of cell death. Instead, ferroptosis may act in concert with microtubule disruption to produce cooperative anticancer effects.

Stimuli-responsive nanocarriers targeting tumor acidity have been widely developed. However, many systems rely solely on pH-triggered degradation or enzymatic cleavage [38, 39]. The current platform incorporates both pH-responsive dissociation and GSH-associated redox modulation. The release data demonstrate minimal drug leakage under physiological conditions and accelerated release in acidic environments, consistent with tumor microenvironment characteristics. In addition, the presence of intracellular GSH potentially facilitates Fe³⁺ reduction, enhancing iron reactivity. Compared with single-trigger systems reported in prior studies, this dual-responsive mechanism may improve spatial selectivity and therapeutic precision [40].

Microtubule disruption leads to G2/M arrest and impaired mitotic progression, as reported in numerous taxane-based studies [41, 42]. Ferroptosis, on the other hand, compromises lipid membrane integrity through iron-dependent peroxidation [43]. These two mechanisms target distinct cellular vulnerabilities: cytoskeletal organization and redox homeostasis. The present findings suggest that simultaneous activation of these stress pathways may reduce the likelihood of adaptive resistance [44, 45]. Previous work has shown that cancer cells with high antioxidant capacity can tolerate chemotherapy-induced ROS [46, 47]. By depleting GSH and suppressing GPX4 while concurrently inducing mitotic stress, Cab@TA–Fe³⁺ may overcome such resistance mechanisms more effectively than monotherapy approaches [36, 48].

Although the in vitro results demonstrate enhanced anti-tumor activity, several limitations remain. First, validation in additional prostate cancer models, including castration-resistant cell lines and in vivo systems, is required. Second, ferroptosis-specific rescue experiments using inhibitors such as ferrostatin-1 would further clarify mechanistic contribution. Third, expanded physicochemical characterization, including drug loading efficiency and colloidal stability in biological media, would strengthen translational potential.

## Conclusion

In summary, our study confirmed the presence of high glutathione and tubulin expression in prostate cancer. On the basis of this results, we developed membrane-coated nanoparticles, a pH/GSH dual-responsive drug delivery system using a tannic acid-Fe³⁺ metal-phenol network to encapsulate Cab, enabling its release through pH-triggered dissociation and GSH-mediated reduction of Fe³⁺ to Fe²⁺ in the tumor microenvironment. The membrane-coated Cab@TA-Fe³⁺ complex demonstrates enhanced anti-tumor efficacy by inducing cytotoxicity, promoting G2/M phase arrest, disrupting microtubule integrity, suppressing cell migration, and promoting death in LNCaP cells, highlighting its potential as a promising therapeutic strategy for targeted tumor treatment. Further, Cab@TA-Fe³⁺ induces ferroptosis by increasing ROS levels, reducing GSH levels, downregulating GPX4 expression, and upregulating NOX1 and PTGS2, thereby amplifying oxidative stress, promoting lipid peroxidation, and enhancing cellular sensitivity to ferroptosis in LNCaP cells. Notably, Ferrostatin-1 partially reversed cytotoxicity and ROS accumulation, indicating that ferroptosis contributes to the therapeutic effect but is not the sole mechanism.Our study presents a novel strategy for prostate cancer treatment that leverages the ferroptosis induced by Fe³⁺ and the microtubule-inhibitory effects of cab, which act in combination to combat prostate cancer.

## Supplementary information

Supplementary information
