# Supplementary material for: Engineered membrane-coated nanoparticles enhance ferroptosis and microtubule inhibition in prostate cancer
Source: J Mater Sci Mater Med. 2026 May 21;37(1):86. doi: 10.1007/s10856-026-07074-7 (PMC13368834; doi:10.1007/s10856-026-07074-7)
Supplement: Supplementary file 1 — Supplementary information [file 10856_2026_7074_MOESM1_ESM.docx]

**Engineered** **Membrane-Coated** **Nanoparticles Enhance Ferroptosis and Microtubule** **Inhibition in Prostate Cancer**

Kunmu Yang^1^, Yingwei Wang^2^, Chuan Guo^1^, Xiaoxiong Zhang^1^, Jian Wu^1*^

^1^Department of Urology, Chengfei Hospital, Chengdu, China

^2^Department of Nuclear Medicine, Affiliated Cancer Hospital of University of Electronic Science and Technology of China, Chengdu, China

Correspondence: Jian Wu (15082758072@163.com)

**Table S1. Primers for RT-qPCR**

| **Primers** | **Primer sequence** | |
| --- | --- | --- |
|  | Forward (5′-3′) | Reverse (5′-3′) |
| **GPX4** | GCCTGGATAAGTACAGGGGTT | CATGCAGATCGACTAGCTGAG |
| **NOX1** | GGTTGGGGCTGAACATTTTTC | TCGACACACAGGAATCAGGAT |
| **PTGS2** | TTCAACACACTCTATCACTGGC | AGAAGCGTTTGCGGTACTCAT |
| **GAPDH** | TCATTGACCTCAACTACATG | TCGCTCCTGGAAGATGGTGAT |
